# Aglycemic HepG2 Cells Switch From Aminotransferase Glutaminolytic Pathway of Pyruvate Utilization to Complete Krebs Cycle at Hypoxia

**DOI:** 10.3389/fendo.2018.00637

**Published:** 2018-10-26

**Authors:** Jan Ježek, Lydie Plecitá-Hlavatá, Petr Ježek

**Affiliations:** Department of Mitochondrial Physiology, Institute of Physiology, Academy of Sciences of the Czech Republic, Prague, Czechia

**Keywords:** cancer mitochondria, HepG2 cells, hypoxia, glutaminolysis, aminotransferase inhibiton, Warburg phenotype

## Abstract

Human hepatocellular carcinoma HepG2 cells are forced to oxidative phosphorylation (OXPHOS), when cultured in aglycemic conditions at galactose and glutamine. These Oxphos cells represent a prototype of cancer cell bioenergetics with mixed aerobic glycolysis and OXPHOS. We aimed to determine fractions of (i) glutaminolytic pathway involving aminotransferase reaction supplying 2-oxoglutarate (2OG) to the Krebs cycle vs. (ii) active segment of the Krebs cycle with aconitase and isocitrate dehydrogenase-3 (ACO-IDH3), which is typically inactive in cancer cells due to the citrate export from mitochondria. At normoxia, Oxphos cell respiration was decreased down to ~15 and ~10% by the aminotransferase inhibitor aminooxyacetate (AOA) or with AOA plus the glutamate-dehydrogenase inhibitor bithionol, respectively. Phosphorylating to non-phosphorylating respiration ratios dropped from >6.5 to 1.9 with AOA and to zero with AOA plus bithionol. Thus, normoxic Oxphos HepG2 cells rely predominantly on glutaminolysis. Addition of membrane-permeant dimethyl-2-oxoglutarate (dm2OG) to inhibited cells instantly partially restored respiration, evidencing the lack of 2OG-dehydrogenase substrate upon aminotransferase inhibition. Surprisingly, after 72 hr of 5% O_2_ hypoxia, the AOA (bithionol) inhibition ceased and respiration was completely restored. Thus in aglycemic HepG2 cells, the hypoxia-induced factor (HIF) upregulation of glycolytic enzymes enabled acceleration of glycolysis pathway, preceded by galactolysis (Leloir pathway), redirecting pyruvate *via* still incompletely blocked pyruvate dehydrogenase toward the ACO-IDH3. Glycolytic flux upregulation at hypoxia was evidently matched by a higher activity of the Leloir pathway in Oxphos cells. Hypoxic Oxphos cells increased 2-fold the NADPH oxidase activity, whereas hypoxic glycolytic cells decreased it. Oxphos cells and glycolytic cells at 5 mM glucose decreased their reduced glutathione fraction. In contrast to aglycemic cells, glycolytic HepG2 cells decreased their respiration at hypoxia despite the dm2OG presence, i.e., even at unlimited respiratory substrate availability for 72 hr at 5% O_2_, exhibiting the canonical HIF-mediated adaptation. Nevertheless, their ATP content was much higher with dm2OG as compared to its absence during hypoxic adaptation. Thus, the metabolic plasticity of cancer cells is illustrated under conditions frequently established for solid tumors *in vivo*, such as aglycemia plus hypoxia. Consequently, a wide acceptance of the irreversible and exclusive Warburg phenotype in cancer cells is incorrect.

## Introduction

During malignant transformation, cells undergo stages of gene expression reprogramming and mutagenesis that alter their metabolic phenotype(s) (1–5). Consequently, dysregulated metabolism is one of the hallmarks of cancer. A partial glycolytic “Warburg” phenotype (aerobic glycolysis) is typically established. This partial glycolytic switch is characterized by the incomplete glucose oxidation stemming from the effective diversion of pyruvate into lactate at the expense of the pyruvate supply into the tricarboxylic acid (Krebs) cycle, hence oxidative phosphorylation (OXPHOS) is decreasing or dormant. Excessive proliferation and impaired angiogenesis cause hypoxia in certain regions within a growing tumor leading typically to stabilization of hypoxia-induced factor-1α (HIF-1 α) and concomitant metabolic reprogramming by the HIF system, including promotion of the aerobic glycolysis (6–10). HIF further intensifies the glycolytic phenotype by the pyruvate kinase muscle isoform (PKM) switch (11) and mitochondrial pyruvate dehydrogenase (PDH) inhibition by PDH kinase-mediated PDH phosphorylation (12, 13).

Despite such inhibition of the pyruvate entry into the Krebs cycle, numerous cancer cell types maintain OXPHOS to a certain extent, notably by glutaminolysis (1–5). This involves glutamine oxidation to glutamate by glutaminase. Glutamate might be oxidized to 2-oxoglutarate (2OG) by glutamate dehydrogenase (GDH), if GDH is not highly inhibited as in typical glutaminolytic cancer cells (1–5). Instead glutaminolysis involves the aminotransferase anaplerotic reaction(s) supplying directly 2OG to the 2OG-dehydrogenase of the Krebs cycle. Alanine aminotransferases (cytosolic ALT1 and mitochondrial ALT2) (also termed glutamate pyruvate transaminases, GPT1 and GPT2) (14) catalyze reversible conversion of pyruvate plus l-glutamate to 2OG and l-alanine (15–21) during glutaminolysis (1–5, 22–25). Analogously, aspartate aminotransferases AST1 and AST2 (also termed glutamate oxaloacetate transaminases, GOT1 and GOT2) convert oxaloacetate plus l-glutamate to 2OG and plus l-aspartate (26, 27). Mitochondrial branched-chain aminoacid aminotransferase (BCAT), present in all tissues, then converts glutamate and a branched-chain 2-oxoacid to a branched-chain amino acid and 2OG (28). Both ALT1 and ALT2 utilize pyruvate for which they compete with lactate dehydrogenase. Without glutaminolysis, there would be a permanent shortage of acetyl-CoA during hypoxia, when the canonical HIF transcriptome reprogramming leads also to inhibition of PDH. If this inhibition was complete, the Krebs cycle and OXPHOS would stop.

In order to ensure the citrate efflux to the cytosol, glutaminolysis allows Krebs cycle to be truncated at the citrate synthase, resulting in unemployed forward aconitase and isocitrate dehydrogenase-3 (ACO-IDH3) activities (1–5). This helps cancer cells to sustain high rate of cell proliferation, since citrate is split to oxaloacetate and acetyl-CoA by the cytosolic ATP-citrate lyase reaction, while the resulting acetyl-CoA serves as a precursor for lipid synthesis. Cytosolic isocitrate dehydrogenase IDH1, malic enzyme, and glucose-6-phosphate (G6P) dehydrogenase reactions provide NADPH within the cytosol, whereas mitochondrial transhydrogenase, malic enzyme, glutamate dehydrogenase, and IDH2 produce NADPH in the matrix (4). Under certain conditions also reductive carboxylation glutaminolysis contributes to the citrate efflux (4), comprising a reverse pathway within the ACO-IDH3 segment, ensured by IDH2 and ACO (22, 29).

The most common liver cancer is hepatocellular carcinoma. This cancer arises from viral infections by hepatitis B and C viruses, alcohol intake, smoking, and many host factors, such as cirrhosis, hemochromatosis, non-alcoholic steatohepatitis, but also obesity and diabetes (30). The metabolic and bioenergetic landscape of human hepatocellular carcinoma has been rarely reported in *in vivo* studies. Hypoxia contributes significantly to carcinogenesis in liver and hepatocellular carcinoma is recognized as one of the most hypoxic tumors with oxygen levels reaching 0.8% (31–33). Despite being understudied, activation of glutaminolysis also significantly contributes to hepatocellular carcinoma development such as during hepatitis C virus infection (34). The nuclear receptor liver receptor homolog 1 (LRH-1) (also known as NR5A2) was recently found to have a regulatory role in hepatoma formation, since it was shown to upregulate mitochondrial ALT2/GPT2 and cytosolic AST1/GOT1 aminotransferase isoforms as well as glutaminase 2 (GLS2) (35).

Despite the existence of reductive carboxylation glutaminolysis in hepatocellular carcinoma HepG2 cells, the HepG2 cell metabolism represents a prototype of predominating OXPHOS glutaminolysis, employing glutaminase followed by the cytosolic ALT1 or matrix ALT2 reaction, both producing 2OG. BCAT can also supply 2OG. The ALT1 and ALT2 aminotransferases require pyruvate, for which they compete with lactate dehydrogenase. If PDH is nearly completely blocked upon hypoxic adaptation, there would be a shortage of acetyl-CoA for citrate synthase and Krebs cycle would be retarded as well as cell respiration.

However, when cultivated with galactose in glutamine-containing media without glucose (“aglycemic” or “Oxphos cells”) (36), HepG2 cells have incompletely phosphorylated PDH, so that ~50 and ~40% of PDH is likely active at normoxia and hypoxia, respectively (37). The non-canonical behavior of these cells can be therefore explained by the incomplete phosphorylation of PDH. In contrast, in HepG2 cells at 5 or 25 mM glucose (glycolytic cells, denoted here as Glc5 cells or Glc25 cells, respectively), only 20% or 5–10% of PDH is active at normoxia and hypoxia, respectively (37). However, despite differential utilization of pyruvate, normoxic respiration is similar for glycolytic and Oxphos cells, indicating that OXPHOS glutaminolysis supports most of the glycolytic cell respiration. Accordingly, hypoxia (72 h at 5% O_2_) let to a ~60% decrease in the rates of respiration and ATP levels in glycolytic but not in aglycemic, i.e., Oxphos cells (37). This was accompanied by the mitochondrial cristae widening, which occurred irrespectively of the carbon source used (38). The unchanged respiration and ATP levels, despite the ongoing HIF-mediated transcriptome reprogramming in the hypoxic aglycemic HepG2 cells was termed as the non-canonical HIF response (37).

Glutaminolysis in aglycemic, i.e., galactose-grown, Oxphos cells coexists with reactions of glycolysis (but the first one converting glucose to G6P), which depend entirely on G6P formed from galactose. The glycolytic pathway supplied by the galactolysis yields three ATP molecules, while one or up to two ATP equivalents are required to match the demand of galactose conversion to G6P (39, 40). Galactolysis is also known as the Leloir pathway and is characterized by a rather low activity (39, 40). All these facts cause that such glycolysis can no longer efficiently supply further energy-requiring processes, neither any high energetic demand. This implies the critical dependence of aglycemic cells on OXPHOS from glutamine. We can refer to it as that these cells are literally forced to OXPHOS. Interestingly, galactolysis has been recently suggested as the prospective therapeutic target for hepatocellular carcinoma, based on inhibition of galactokinase (GALK) or galactose-1 phosphate uridylyltransferase (GALT) (40). Also this finding prompted us to investigate whether the upregulation of the glycolytic flux at hypoxia can be matched by a higher activity of the Leloir pathway in model HepG2 cells.

Cancer cells can also use fatty acids (FAs) derived from the hydrolysis of triglycerides found in either lipid droplets, if exist, or following their uptake from the extracellular environment. Lipophagy, a specific autophagic pathway, participates in the process of lipid hydrolysis (41). Malignant tumor cells, including hepatocellular carcinoma (42), upregulate NADPH-dependent FA synthesis for building their membrane constituents and signaling molecules (43). In many tumors, this is coordinated by the oncoprotein c-Myc (44). Besides aerobic glycolysis, citrate can be also generated by glutaminolysis for subsequent ATP citrate lyase reaction providing acetyl-CoA. Fatty acid synthase (FASN) upregulated by human epidermal growth factor receptor 2 (Erb-B2) (also known as HER/neu), a tyrosine kinase receptor, is frequently overexpressed in cancer cells, which promotes their invasiveness and proliferation (4). In conclusion, lipids are crucial for proliferation, growth, survival, invasion, and angiogenesis in cancer cells (45).

In contrast, FA oxidation is frequently inhibited in tumors (42, 46). However, upon metabolic stress, FA oxidation is re-established to sustain ATP synthesis (47, 48) and NADPH formation (49). Thus, activation of FA oxidation may lead to lower proliferation rate and serve as a marker of improved prognosis (50). Aglycemia shifts metabolism toward NADPH formation by activation of the liver kinase B1 (LKB1)/AMP-activated protein kinase (AMPK) pathway. This pathway is responsible for the balance between FA synthesis (i.e., NADPH consuming reactions) and NADPH-producing reactions, including FA oxidation.

Studying extreme conditions of aglycemia/hypoxia, while inhibiting aminotransferases in HepG2 cells, we found a metabolic switch in hypoxia-adapted aglycemic cells (forced to OXPHOS) from glutaminolysis to the forward Krebs cycle including aconitase-IDH3 reactions. Thus, partial glycolysis elevation in aglycemic hypoxic cells, matched by elevated galactolysis, induced higher metabolic fluxes *via* the ACO-IDH3 segment and maintained OXPHOS levels during such a non-canonical HIF response. In contrast, when glucose was available, i.e., for glycolytic cells, the typical HIF-mediated adaptation occurred, resulting in partly dormant OXPHOS. Such adaptation existed even at the unlimited substrate availability during hypoxic adaptation. Nevertheless, the latter led to higher ATP levels. In conclusion, we clearly demonstrate a high metabolic plasticity of hepatocellular carcinoma HepG2 cells, dependent on oxygen and glucose availability.

## Materials and methods

### Materials

Aminooxyacetate (AOA, *O*-(carboxymethyl) hydroxylamine hemihydrochloride) and other reagents were from Sigma-Aldrich (St. Louis, MO), unless stated otherwise.

### Cell culture

Human hepatocellular carcinoma (hepatoma) HepG2 cells (ECACC, Salisbury, UK, 85011430) were cultured at 37°C in a humidified incubator with 5% CO_2_ in glucose-free DMEM (Thermo Fisher Scientific, Waltham, MA) supplemented with 3 mM glutamine, 10% (v/v) fetal calf serum (Thermo Fisher Scientific), 10 mM HEPES, 100 IU/ml penicillin and 100 μg/ml streptomycin, and with either 5 mM glucose (denoted here as Glc5 cells), 25 mM glucose (Glc25 cells), or 10 mM galactose (Oxphos cells) (37, 38). Galactose-grown Oxphos cells were cultured in a glucose-free, dialyzed fetal calf serum (Biochrom AG, Berlin, Germany). Hypoxia was set in a SCI-tive N workstation (Ruskinn Technology, Bridgend, UK) with 5% O_2_ (~40 mm Hg; O_2_ replaced by N_2_) and 5% CO_2_. Before each experiment, cells were detached by trypsinization. Aliquots of such suspension were stained with trypan blue and counted for viable (trypan blue-negative) cells.

### High-resolution respirometry

Cellular O_2_ consumption was measured in the culture medium using an Oxygraph-2k (Oroboros, Innsbruck, Austria) after air calibration and background correction (37). Routine cell respiration reflects phosphorylating respiration (termed state 3 in isolated mitochondria with saturated ADP). Non-phosphorylating state (termed state 4 in isolated mitochondria) was induced by 0.1 μg/ml oligomycin (37). Maximum uncoupled respiration rates were determined after titration with the uncoupler, carbonyl cyanide-p-trifluoromethoxyphenylhydrazone (FCCP) at around 1 μM. To determine O_2_ consumption during hypoxia, oxygen was replaced with nitrogen to reach 5% O_2_ levels prior to cell addition. Doses of all inhibitors used were derived from titrations after achieving nearly saturation concentrations.

### Confocal microscopy monitoring of mitochondrial membrane potential

Cells were cultured for 2 days on glass coverslips coated with poly-l-lysine. A TCS SP2 AOBS confocal microscope (Leica Microsystems, Mannheim, Germany) was used with a PL APO 100 × /1.4–0.7 oil immersion objective (pinhole, 1 Airy unit) and a thermostable sample chamber (37°C) supplied with 5% CO_2_ and either atmospheric air or humidified N_2_ and 5% O_2_. Note that this parameter is an ambient value, since the effective oxygen tension established at the cell layer seeded onto bottom of a culture flask is lower. Monitoring of mitochondrial membrane potential Δψ_*m*_ was done using the ratiometric JC-1 probe (Thermo Fisher Scientific) [see Supplementary material in JeŽek et al. (51)]. Excitation was set at 488 nm, and emissions were collected between 500 and 600 nm under a confocal microscope. Ratio of JC-1 fluorescence at 593 vs. 537 nm has been shown previously to fall into a range of 0.2 to 0.4 in our experimental setup, the former value corresponding to the lowest potential near zero and the latter value to the high Δψ_*m*_.

### Quantification of lactate, ATP, superoxide dismutase, and the ratio of reduced to oxidized glutathione

A Lactate Assay Kit (BioVision, Milpitas, CA), ATP Bioluminescence Assay Kit HSII (Roche, Basel Switzerland) were used. To quantify superoxide dismutase (SOD), an SOD Activity Assay Kit (Cayman, Ann Arbor, MI) was used, while KCN additions were also made to quantify specifically the MnSOD activity. A Glutathione Assay Kit (BioVision, Milpitas, CA) was used to quantify reduced (GSH), oxidized (GSSG), and total glutathione, hence allowing calculations of the GSH/GSSG ratio.

### Analysis of NADPH oxidase activity

A nitroblue tetrazolium (NBT) reduction assay was employed to estimate NADPH oxidase (NOX) activity. Trypsinized cells were freeze-thawed three times, and hence permeabilized, in 100 mM Tris-HCl (pH 7.4), followed by the addition of 2 mM NBT and 1 mM NADPH. The resulting blue formazan was detected at 530 nm using a spectrophotometer (Olis, Bogart, GA).

### Monitoring of reduced NAD(P)H/FAD

Cell autofluorescence, as a measure of the NAD(P)H to FAD ratio, was monitored on an RF5301PC spectrofluorometer (Shimadzu, Tokyo, Japan) [excitation, 350 nm; emission, 460 nm for NAD(P)H and excitation, 450 nm; emission, 535 nm for FAD, at 10 nm wide slits].

### Western blotting

Proteins were extracted by cell lysis in 50 mM HEPES, 150 mM NaCl, 1 mM EDTA, 1% Triton TX-100, 1 mM phenylmethylsulfonyl fluoride, pH 7.4, and protein content was quantified using bicinchoninic acid (BCA) assay (Pierce, Rockford, IL). Proteins separated by sodium dodecyl sulfate-polyacrylamide gel electrophoresis (SDS-PAGE) were transferred by semidry electroblotting onto polyvinylidene difluoride (PVDF) membranes, treated with the primary and horseradish peroxidase-conjugated secondary antibodies. Enhanced Chemiluminescence (ECL) detection was accomplished using Amersham blotting kit (GE Healthcare Bio-Sciences Corp., Piscataway, NJ) with monoclonal antibodies against HIF-1α (BD Biosciences, San Jose, CA). The ECL light intensity was quantified by densitometry using the Scion Image software (beta 4.02 Win).

### Statistical analysis

Analysis of variance (ANOVA) was carried out using SigmaStat 3.1 (Systat Software, San Jose, CA), was carried out with subsequent pairwise multiple comparisons (Tukey *post-hoc* test).

## Results

### Hypoxic OXPHOS cells switch from aminotransferase-dependent glutaminolysis to galactose utilization and complete krebs cycle

**Figure 1:**
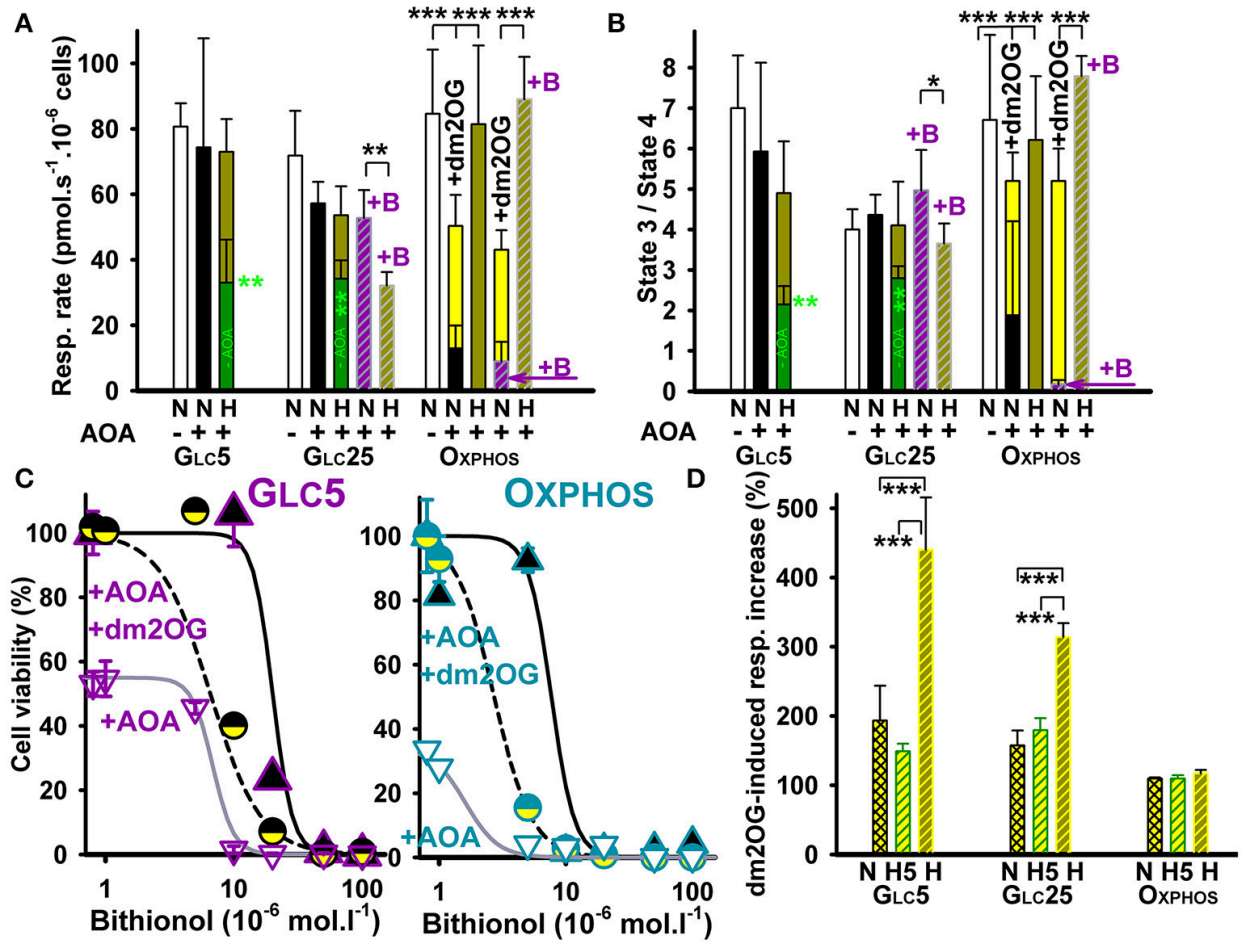
HepG2 cell respiration and viability at inhibited aminotransferases and glutamate dehydrogenase**. (A)** Respiratory rates (mean ± s.d.) are shown for 5–7 independent cell cultures for each metabolic regime, assayed in a total of 5–14 experimental runs for each condition. **(B)** phosphorylating/non-phosphorylating respiration ratio (“State 3/state 4”) was calculated from rates in **(A)** and those at state 4 induced by 0.1 μg/ml oligomycin. Values are shown for normoxic cells (“N”) without additions (*white bars*), with AOA (*black bars*), or with AOA plus bithionol (“+B”) (*dashed purple bars)*. This is compared with values for hypoxic cells adapted for 72 hr at 5% oxygen (“H”) without additions (*dark green bars*), with AOA (*yellow green bars*), or with AOA plus bithionol (“+B”) (*dashed yellow green bars*). Treatments with 0.5 mM AOA or with 0.5 mM AOA plus 0.5 μM bithionol were performed during the last 24 hr of hypoxic treatment. *Yellow bars*: 4 mM dm2OG added prior to the assay. ANOVA: ^*^p < 0.1; ^**^p < 0.05; ^***^p < 0.001. **(C)** Toxicity of bithionol with or without AOA and its rescue by dm2OG – Cell viability is shown for normoxic Glc5 (*left panel*) and Oxphos cells (*right panel*). The derived LD_50_ values were 20, 6, and 6 μM for Glc5 cells and 9, 1.1, and 2.5 μM for Oxphos cells treated with bithionol alone, bithionol with 0.5 mM AOA and bithionol with 0.5 mM AOA and 4 mM dm2OG, respectively. **(D)** Relative instant increase in respiration induced by dm2OG (4 mM, *yellow*) is shown for each metabolic state prior to (“N”)(*crossed yellow bars*) and after a 5 hr hypoxia (“H5”)(*dashed yellow bars*) and 72 hr hypoxia (“H”), (*dashed yellow-green bars*). Values are normalized to normoxic Oxphos cells. Hypoxic treatment was at 5% oxygen in the absence of dm2OG. ANOVA (*n* > 4): ^***^p < 0.001.

We cultured HepG2 cells under three distinct metabolic regimes, setting the different relative contributions of sole (aerobic) glycolysis and OXPHOS (plus glycolysis) to energy (ATP) production. In all cases, a mixed OXPHOS/aerobic glycolysis metabolism occurs, while respiration amounts to 70–80 pmol per second and milion cells (Figure 1A) (37). In glycolytic cells cultivated with glutamine and either at 5 mM glucose (Glc5 cells) or at 25 mM glucose (Glc25, hyperglycemic cells), highly phosphorylated PDH limits the pyruvate—acetyl-CoA and the ACO-IDH3 segment of the Krebs cycle, which is nearly eliminated by the concomitant citrate export from the matrix. OXPHOS is supplied by 2OG derived from glutaminolysis and coexists with aerobic glycolysis. Oxphos cells cultivated with glutamine plus galactose also exhibit OXPHOS from glutaminolysis and since PDH is at least ~50% functional (37), the pyruvate—acetyl-CoA input into the Krebs cycle may be active.

**Figure 2:**
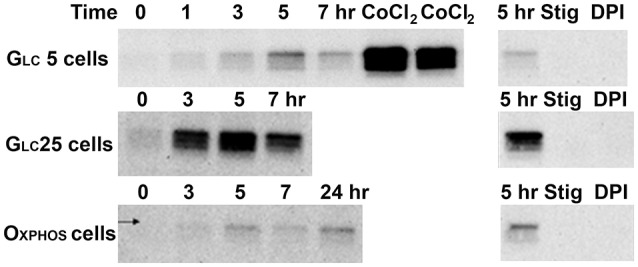
Hypoxic and CoCl_2_-induced normoxic HIF1α stabilization. HIF1α content (protein-normalized Western blots)-Left panels: HIF1α content after the indicated time at 5% O_2_ or 24-h incubation with 100 μM CoCl_2_ “CoCl_2_”; Right panels: 5-h incubation at 5% O_2_ without any agent “5 h” or with 0.5 μM stigmatelin “Stig” or with 10 μM diphenyleneiodonium chloride (“DPI”).

A 72 hr 5% O_2_ hypoxic adaptation was routinely performed, leading to HIF-1α maximum stabilization at the 5th hr for all three metabolic regimes (Figure 2, left panels). HIF-1α stabilization was completely prevented when respiratory chain Complex III was inhibited by stigmatellin or Complex I by diphenyleneiodonium (Figure 2, left panels) and was also evoked at normoxia by CoCl_2_.

To estimate a fraction of metabolic flux bypassing the PDH reaction and the ACO-IDH3 segment by parallel glutaminolytic aminotransferase reaction, aminotransferases were blocked by their specific inhibitor aminooxyacetate (AOA) (26, 52). If glutamate dehydrogenase (GDH) was blocked, AOA would stop the glutaminolytic 2OG supply to the Krebs cycle. The resulting respiration should then originate from the remaining metabolic fluxes of the sole glycolytic/pyruvate—acetyl-CoA plus ACO-IDH3 axis. Normoxic AOA incubations (during the last 24 hr of total 72 hr) decreased cell phosphorylating respiration to ~15% (Figure 1A) and phosphorylating/non-phosphorylating respiration ratio dropped from >6.5 to 1.9 (Figure 1B) in Oxphos cells. The latter reflects the intensive OXPHOS decline. The respiration decrease accounted for ~85% of glutaminolysis contribution in normoxic Oxphos cells.

Surprisingly, hypoxic incubations at 5% O_2_ with AOA (during the last 24 hr of total 72 hr) did not significantly inhibit either Oxphos cell respiration (Figure 1A, yellow green bars) or phosphorylating/non-phosphorylating ratios (Figure 1B). Additional 0.5 μM bithionol, the highly specific GDH inhibitor (53), together with AOA, reduced normoxic Oxphos cell phosphorylating respiration even further down to ~10% (Figure 1A), while phosphorylating/non-phosphorylating ratio approached to zero (Figure 1B). In contrast, hypoxic incubations were not affected (Figures 1A,B). The AOA- or AOA plus bithionol-suppressed normoxic respiration and OXPHOS were instantly partially restored in all cases by the addition of membrane-permeant 2OG analog, dimethyl 2-oxoglutarate (dm2OG) (yellow bars in Figures 1A,B). This instant partial recovery reflects the dormant character of OXPHOS due to a 2OG shortage.

Our data reflect up to 90% dependency of Oxphos cells on glutaminolysis prior to hypoxic adaptation and the existence of a switch upon the hypoxic adaptation toward the regular canonical PDH plus Krebs cycle pathway including the ACO-IDH3 segment. Redirection of pyruvate is supported by our previous finding of very low lactate formation upon normoxia and hypoxia in Oxphos cells (37). This switch is able to substitute the metabolic flux (equivalent to a 2OG flux utilized by 2OGDH) lost due to the inhibited aminotransferases and/or GDH. The concomitant HIF-dependent upregulation of glycolytic enzymes in Oxphos cells and only a partial PDH blockage from normoxic ~50% to only ~60% at hypoxia cannot prevent such redirection of pyruvate. Galactolysis is evidently able to supply fast enough G6P to the upregulated glycolysis followed by the complete Krebs cycle and OXPHOS. In conclusion, hypoxia enables redirection of all pyruvate metabolic flux in mitochondria to the canonical Krebs cycle as suggested by the resulting flux insensitivity to the aminotransferase and GDH inhibitor(s) (Figures 1A,B).

AOA did not significantly alter respiration and phosphorylating/non-phosphorylating respiration ratio in normoxic glycolytic Glc5 cells and reduced respiration only slightly in normoxic Glc25 cells (Figures 1A,B). Hypoxic glycolytic Glc5 and Glc25 cells decreased their respiration down to ~40%, whereas AOA partly prevented this decrease (applied for last 24 hr during 72 hr at 5% O_2_). With AOA plus bithionol the partial respiration blockage by ~50% remained in Glc25 cells (Figure 1A). These results indicate the inherent plasticity of glycolytic cells, alternating readily between the glutaminolytic and regular Krebs cycle pathway. The remaining very low PDH capacity has to be supported by other anaplerotic reactions to be able to keep high respiration after hypoxic adaptation at inhibited aminotransferases, namely ALT2. Note also that with 0.5 mM AOA at bithionol concentrations >10 μM both normoxic glycolytic (Figure 1C) and normoxic Oxphos cells died (Figure 1D). Interestingly, co-incubations with dm2OG predominantly prevented such cell death (Figures 1C,D), demonstrating again importance of the glutaminolysis at normoxia.

### Dormant mitochondria in hypoxic glycemic cells possess substrate shortage

Further instant dm2OG additions to normoxic Oxphos cell did not elevate their respiration, neither when these instant additions were done after their hypoxic adaptation. (Figure 1D). In contrast, instant dm2OG additions to the normoxic and hypoxia-adapted glycolytic cells for 5 hr raised instantly their otherwise down-regulated endogenous respiration by ~2-fold. Respiration was instantly raised upon such dm2OG addition even by 4- and 3-fold in Glc5 and Glc25 cells, respectively, after 72 hr of hypoxia (Figure 1D). This reflects the sufficient capacity for mitochondrial ATP production in glycolytic cells even prior to their adaptation and excessive after the adaptation. After hypoxic adaptation, OXPHOS is dormant in glycolytic cells due to a ~95% PDH blockage (37), while aerobic glycolysis predominates. However, the intactness and readiness to function is clearly recognized for the OXPHOS machinery in glycolytic cells, since they respond immediately to sudden respiratory substrate availability. In conclusion, Warburg phenotype in hypoxic glycolytic cells is instantly partially released by dm2OG addition.

### Mitochondrial membrane potential slightly decreases in hypoxia

**Figure 3:**
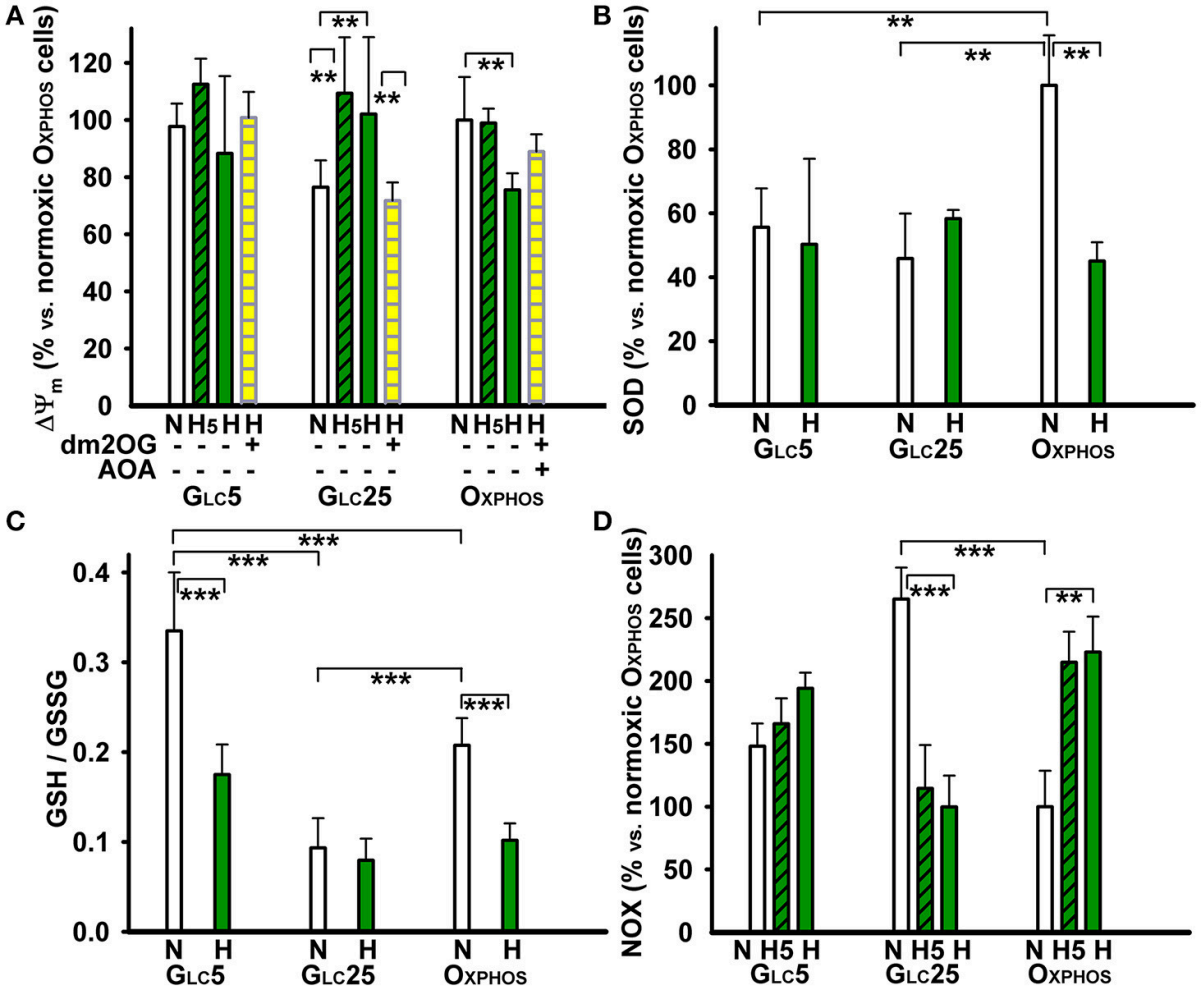
Other metabolic parameters of HepG2 cells at atmospheric and 5% oxygen–**(A)** Δψ_*m*_ was assessed by the JC-1 ratiometric fluorescence probe. **(B)** The total SOD activity, **(C)** ratio of reduced glutathione to oxidized glutathione, and **(D)** NOX activity, were assayed as described in the Materials and Methods. Glc5 and Glc25 glycolytic cells and aglycemic Oxphos cells were cultured in normoxic conditions (“N”) or hypoxia-adapted at 5% O_2_ (*dark green*) for 5 hr (“H5”) or 72 hr (“H”). The culture medium included 4 mM dm2OG (instant addition; *yellow*) and 0.5 mM AOA as indicated. Data in **(A,B,D)** are shown relative to normoxic Oxphos cells; data in **(C)** refer to absolute magnitudes. ANOVA, Tukey's test (*n* > 4): ^***^p < 0.001; ^**^p < 0.05.

The electric component of the protonmotive force, the membrane potential Δψ_m_, established on the inner mitochondrial membrane, declined in Oxphos cell after 72 hr of hypoxia, but not after 5 hr, when HIF-1α stabilization is peaking (Figures 2, 3A). The dm2OG addition (together with AOA) to hypoxia-adapted Oxphos cells did not significantly alterΔψ_m_. Likewise Δψ_m_ was not significantly different after 72 hr hypoxic treatment of Glc5 cells, neither with dm2OG. Hyperglycemic Glc25 cells exhibited increased Δψ_m_ after 5 hr of hypoxia, which remained equal after 72 hr of hypoxia and which declined when dm2OG was present during the assay (Figure 3A).

### Antioxidant capacity in normoxia vs. hypoxia

The total superoxide dismutase (SOD) activity was twice as high in normoxic Oxphos cells when compared to both types of glycolytic cells (Figure 3B). After a 72 hr hypoxic adaptation at 5% O_2_ the total SOD activity of Oxphos cells dropped to about half, down to levels in glycolytic cells, in which the total SOD activity was unchanged at hypoxia. The overall redox state, as judged from the GSH/GSSG ratio, was reduced to maximum in Glc5 cells, followed by Oxphos cells. Nevertheless, this ratio was 3-fold lower (the most oxidized) in Glc25 cells (Figure 3C). After 72 hr at 5% O_2_, the GSH/GSSG ratio significantly dropped to about half in Oxphos and Glc5 cells, indicating that hypoxia established more oxidized state; while the already normoxic oxidized state of hyperglycemic glycolytic Glc25 cells was not further oxidized (Figure 3C).

### NADPH oxidase activity and NAD(P)H/FAD ratios at normoxia vs. hypoxia

**Figure 4:**
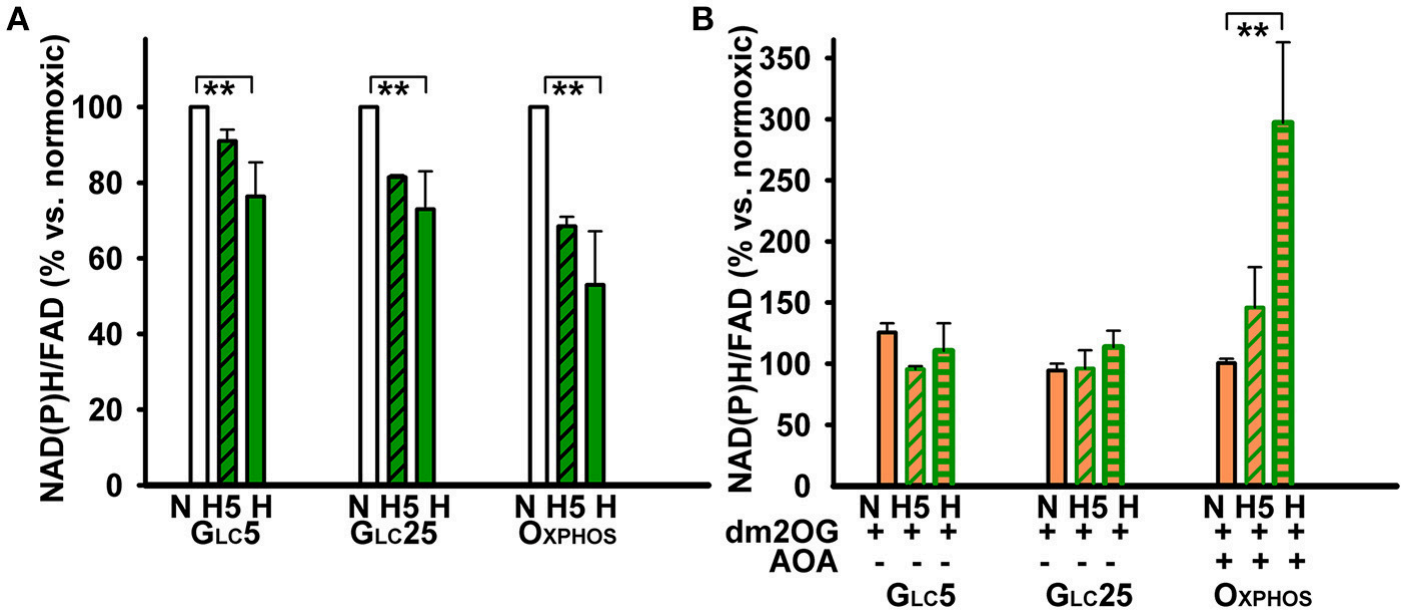
NAD(P)H/FAD ratios were determined in the absence **(A)** and presence **(B)** of 4 mM dm2OG (*orange*) during normoxic or hypoxic incubations (*dark green*) and normalized relatively to the normoxic condition for each metabolic cell type. For other description see the Legend to Figure 2.

Oxphos cells increased 2-fold their NADPH oxidase (NOX) activity at hypoxia already beginning from the 5th hr of adaptation at peaking HIF-1α, in contrast to Glc5 cells, which increased it insignificantly (Figure 3D). Glc25 cells exhibited high NOX activity already at normoxia at atmospheric O_2_ levels. This activity again decreased beginning from the 5th hr of hypoxic adaptation to 5% O_2_ down to the levels found in normoxic Oxphos cells. Next, we inspected, whether the mitochondrial plus NOX activity is reflected by the overall NAD(P)H/FAD ratios under the studied conditions. These ratios declined to half for Oxphos cells and only by ~30% in glycolytic cells, while intermediate levels were found at the 5th hr of hypoxic adaptation (Figure 4A). Interestingly, at the unlimited respiratory substrate availability during hypoxic adaptation (proceeding in the dm2OG presence) the drop in NAD(P)H/FAD ratios was eliminated. The NAD(P)H/FAD ratios even increased in Oxphos cells despite AOA was present together with dm2OG starting from the 5th hr of hypoxic adaptation (Figure 4B). This again shows that anaplerotic reactions are feeding the Krebs cycle despite the HIF-mediated PDH inhibition.

### Cell respiration after hypoxic adaptation with an unlimited substrate availability

**Figure 5:**
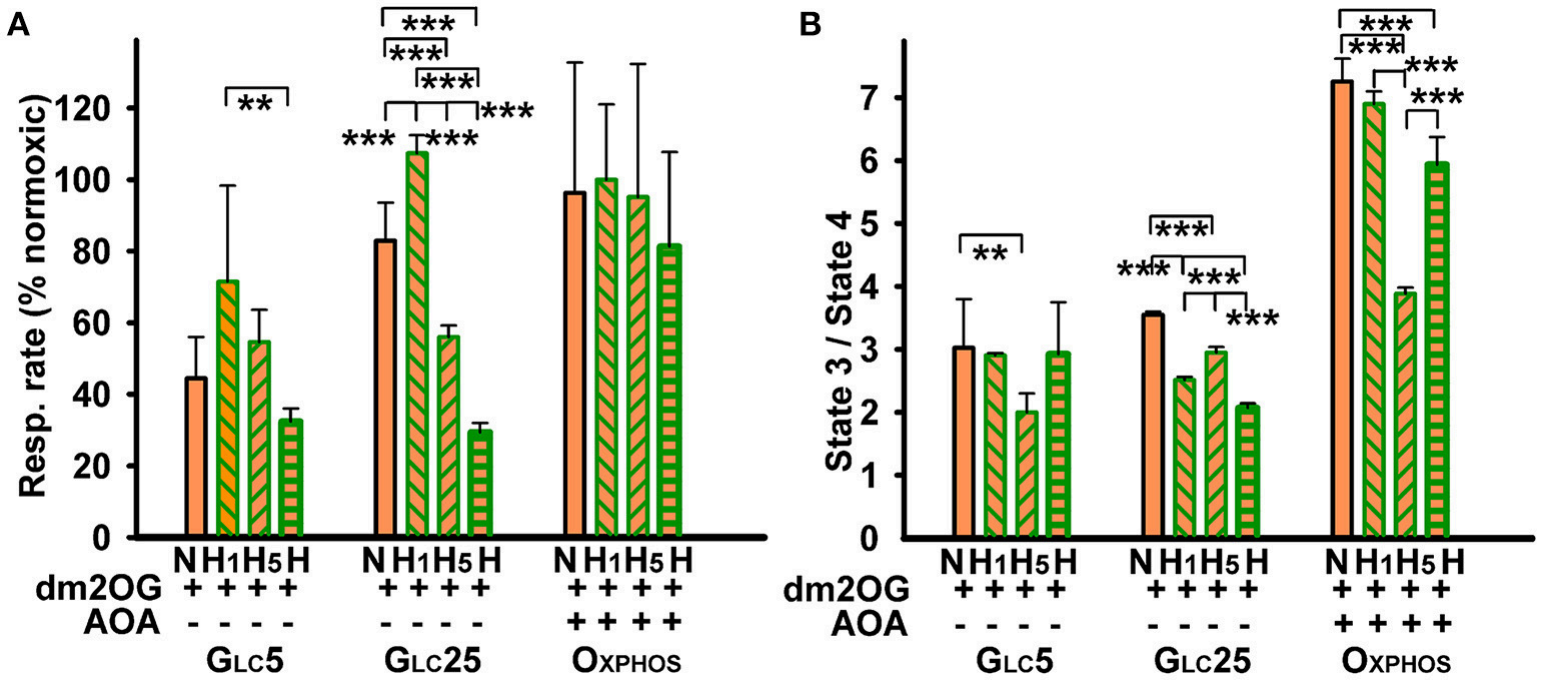
Effect of dm2OG preincubation on cell respiration: cells were incubated for 72 hr at normoxia (“N”) or at hypoxia (*dark green*) for 1 h (“H1”), (“H5”), or 72 h (“H”) with 4 mM dm2OG (*orange*) (0.5 mM AOA was included with the dm2OG for Oxphos cells), and then respiratory rates were determined. The respiratory rates are normalized to normoxic rates in the absence of dm2OG **(A)** and state-3/state-4 respiration ratios **(B)** were calculated, while the state-4 was induced by 0.1 μg/ml oligomycin. Data (mean ± s.d.) are present 5–7 cultivations of each cell type, including altogether 5–14 assays for each condition. ANOVA, Tukey's test: ^***^p < 0.001; ^**^p < 0.05.

We have also quantified respiration of cells adapted to hypoxia in the presence of dm2OG. This membrane-permeable 2OG analog, which is de-methylated in the cytosol thus releasing 2OG, imposes an unlimited substrate supply during hypoxic adaptation. Respiration of Oxphos cells was virtually unchanged with the regards to the atmospheric conditions and despite the additional presence of AOA (Figure 5A). This result complies with the previous findings of such a non-canonical response to HIF (37) and also shows the pathway saturation already existing at normoxia. Nevertheless, as concerning the phosphorylating/non-phosphorylating respiration ratio, it significantly dropped at the 5th hr of hypoxic adaptation of Oxphos cells at 5% O_2_, but returned intermediately to higher values after 72 hr at 5% O_2_ (Figure 5B). We conclude that OXPHOS is preserved despite the hypoxia in aglycemic cells.

Similarly as in the absence of dm2OG (Figure 1A), hypoxic glycolytic cells decreased their respiration (Figure 4A) leaving dormant OXPHOS with much lower phosphorylating/non-phosphorylating respiration ratio after 72 hr with dm2OG at 5% O_2_ (Figure 5B). Thus, HIF-mediated OXPHOS downregulation proceeded despite the dm2OG presence. Nevertheless, at the initial adaptation phases, dm2OG increased respiration of glycolytic cells, such as seen after 1 hr at 5% O_2_. This reflects the response to such substrate surplus in not yet hypoxia-adapted cells. However, respiration declined already at the 5th hr of hypoxic adaptation (Figure 5A). The OXPHOS intensity taken as the phosphorylating/non-phosphorylating respiration ratio occurred to be maintained at the same but 2-fold lower levels when compared to Oxphos cells and declined even more in Glc25 cells. Hyperglycemia together with hypoxia thus sets the lowest OXPHOS intensity.

### ATP levels at normoxia vs. hypoxia

**Figure 6:**
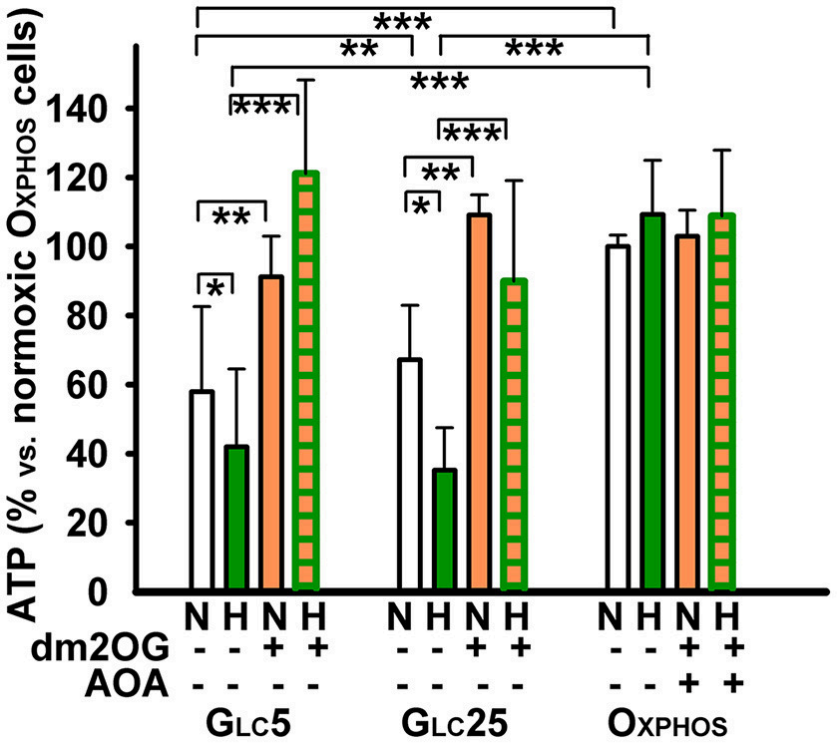
Cellular ATP levels, normalized to values for normoxic Oxphos cells which were set as 100%. Cells were cultured with (*orange*) and without 4 mM dm2OG as indicated. At normoxia (“N”), dm2OG incubatiosn lasted only 10 min; otherwise dm2OG was present during the whole 72-hr hypoxic incubation at 5% O_2_ (*dark green*)(“H”); 0.5 mM AOA was included with the dm2OG for Oxphos cells. ANOVA, Tukey's test (*n* = 4): ^*^p < 0.1, ^**^p < 0.05, and ^***^p < 0.001.

Next, we intended to confirm conclusions made on the basis of respiration assay by the direct estimation of the total ATP content. Note that both glycolysis and OXPHOS contribute to the final measured ATP levels. Oxphos cells did not change their ATP levels after the 72 hr hypoxic adaptation at 5% O_2_ and maintained them despite the presence of dm2OG (together with AOA) during this adaptation (Figure 6). The ATP content at normoxia was lower in glycolytic cells accounting for 60 and 70% of Oxphos cell levels in Glc5 and Glc25 cells, respectively (Figure 6). Complying with the decreasing respiration, the ATP content was depleted by ~30 and ~50% in Glc5 and Glc25 cells (as related to their normoxic values), respectively, after 72 hr hypoxic adaptation to 5% O_2_. However, when dm2OG was present during the hypoxic adaptation ATP levels returned to the maximum values found in Oxphos cells (Figure 6).

## Discussion

Atmospheric cultivation is regarded here as “normoxic,” despite being in fact hyperoxic, when compared to the physiological environment and oxygen tension in the liver (31, 54, 55). Nevertheless, the term normoxia here is describing the standard cell cultivation conditions at atmospheric O_2_. The conducted mild hypoxic adaptations at 5% O_2_ are then related to these standard conditions. Undoubtedly the intermittent periods of aglycemia-hypoxia exist in solid tumors *in vivo* (31, 55). Typical cancer cells with the established aerobic glycolysis (Wargburg phenotype) can survive these aglycemic periods only by its substitution by glutaminolysis relying on OXPHOS. On the other hand, glutamine shortage restores intensive glycolytic metabolism, but cannot restore OXPHOS (54).

**Figure 7:**
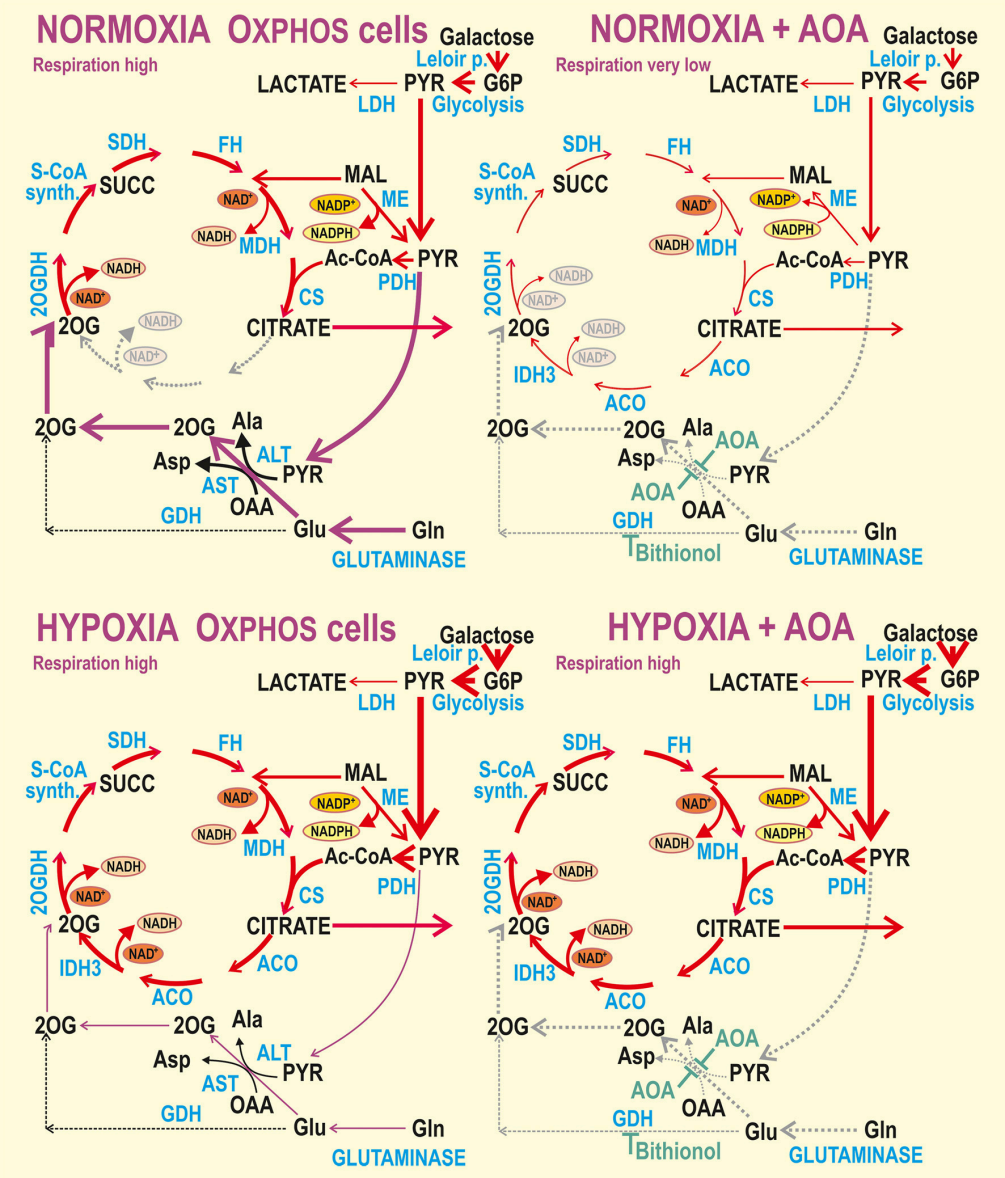
Schemes of normoxic *vs*. hypoxic metabolism for aglycemic (Oxphos) cells without or with inhibited aminotransferases and GDH. Interpretation of the obtained data is illustrated by thicker arrows representing higher metabolic fluxes or gray dotted arrows representing interrupted or inhibited fluxes. For simplicity, the BCAT reaction is not marked and the names of most metabolites are omitted. BCAT would convert glutamate and either 2-oxoisovalerate or 2-oxoisocaproate or 2-oxo-3-methylvalerate to 2OG and valine, leucine or isoleucine, respectively (28). ACO, aconitase; CS, citrate synthase; FH, fumarate hydratase; GDH, glutamate dehydrogenase; IDH3, isocitrate dehydrogenase isoform 3; LDH, lactate dehydrogenase; MDH, malate dehydrogenase; ME, malic enzyme; 2OGDH, 2-oxoglutarate dehydrogenase; PDH, pyruvate dehydrogenase; SCoA synth., succinyl-coenzyme A synthase; SDH, succinate dehydrogenase (Complex II of the respiratory chain).

We now show that cancer cell metabolic plasticity is substantiated by a shift of aglycemic hypoxic hepatocellular carcinoma HepG2 cells from the predominant glutaminolysis at normoxia to the complete Krebs cycle (Figure 7), following hypoxic adaptation of a non-canonical type, which preserves OXPHOS. Such a non-canonical HIF response at aglycemia, rather than down-regulation of oxidative phosphorylation, was previously identified resulting from the incomplete PDH phosphorylation/inhibition at aglycemia (37). Despite we are aware of the limitation when using high resolution respirometry in conjunction with enzyme inhibitors for interrogating changes in metabolic circuits without the use of metabolomics, the derived conclusions and the demonstration of metabolic flexibility in HepG2 cells are relevant. The use of inhibitors contributes to flux repartition. Our findings are consistent with the expected effect of the different inhibitors used. Moreover, the use of aminotransferase inhibitor AOA is versatile, since multiple genetic ablations would be necessary to mimic their systemic effects. Combination of chemical AOA inhibition and its rescue by dm2OG has been described as highly specific (25).

OXPHOS glutaminolysis is essential for cancer cell survival, since during aglycemia, glutamine becomes the main alternative carbon source (4, 22–25, 37, 56). We have previously demonstrated that in hypoxia-adapted Oxphos cells, conversion of pyruvate to acetyl-CoA is active during aglycemia, since PDH is incompletely phosphorylated (37). The tricarboxylic acid cycle, termed here as the Krebs cycle, then acts efficiently providing intermediates for the respiratory chain and ATP synthesis. For all HepG2 metabolic modes, we confirmed that hypoxic adaptation elevates glycolysis, presumably *via* glycolytic enzyme upregulation by the HIF system. This was valid also for the galactose-driven glycolysis in Oxphos cells. We demonstrated that even at aglycemia hypoxic adaptation is so intensive, that enables more rapid pyruvate production, which is subsequently used by aminotransferases, namely by ALT2 (Figure 7) for anaplerosis, i.e., 2OG supply to the Krebs cycle in parallel with glutamine utilization. Despite the partly (nearly completely in glycolytic cells) suppressed pyruvate entry *via* PDH, pyruvate still promotes respiration due to the ALT2 reaction, forming alanine and 2OG from glutamate as a co-substrate (4). Alternatively, mitochondrial BCAT, present in all tissues, may convert glutamate and a branched-chain 2-oxoacid to a branched-chain amino acid and 2OG (28). Overall, the Krebs cycle maintains its turnover and virtually unchanged respiration (or decreased but non-zero in glycolytic cells). In this way, a hypoxic blockage of PDH is compensated for anaplerosis.

The galactolysis by the Leloir pathway (39, 40, 57) is evidently able to supply G6P to the HIF-enhanced glycolysis, which is followed by pyruvate and acetyl-CoA production and OXPHOS in hypoxic Oxphos cells. Even with no glucose in external media, the intracellular glucose derivatives are formed. The ongoing glycolysis from galactose is dependent on phosphoglucomutase, converting glucose-1-phosphate to G6P (39, 40). Phosphoglucomutase is the last enzyme of the Leloir pathway, which is initiated by GALK phosphorylating galactose to galactose-1-phosphate. This reaction requires one ATP molecule. Galactose-1-phosphate reacts with UDP-glucose to form UDP-galactose and glucose-1-phosphate in a reaction catalyzed by GALT (39, 40, 57). UDP-glucose is recycled from UDP-galactose by UDP-galactose 4'-epimerase (GALE) for the next round of GALT reaction (39, 40). Both GALK and GALT have recently been identified as prospective therapeutic targets in hepatocellular carcinoma (40). Also UDP-glucose pyrophosphorylase provides UDP-glucose from glucose-1-phosphate and UTP, an ATP equivalent. Since glycolysis from G6P produces 3 ATP molecules, the net energy balance of galactolysis followed by glycolysis from G6P is either two or one ATP molecules. Due to this fact and rather a slow kinetics of the Leloir pathway (39, 40), the aglycemic cells would die unless OXPHOS relying on glutaminolysis is not producing further ATP. That is why we describe this situation as cells “forced” to OXPHOS.

**Figure 8:**
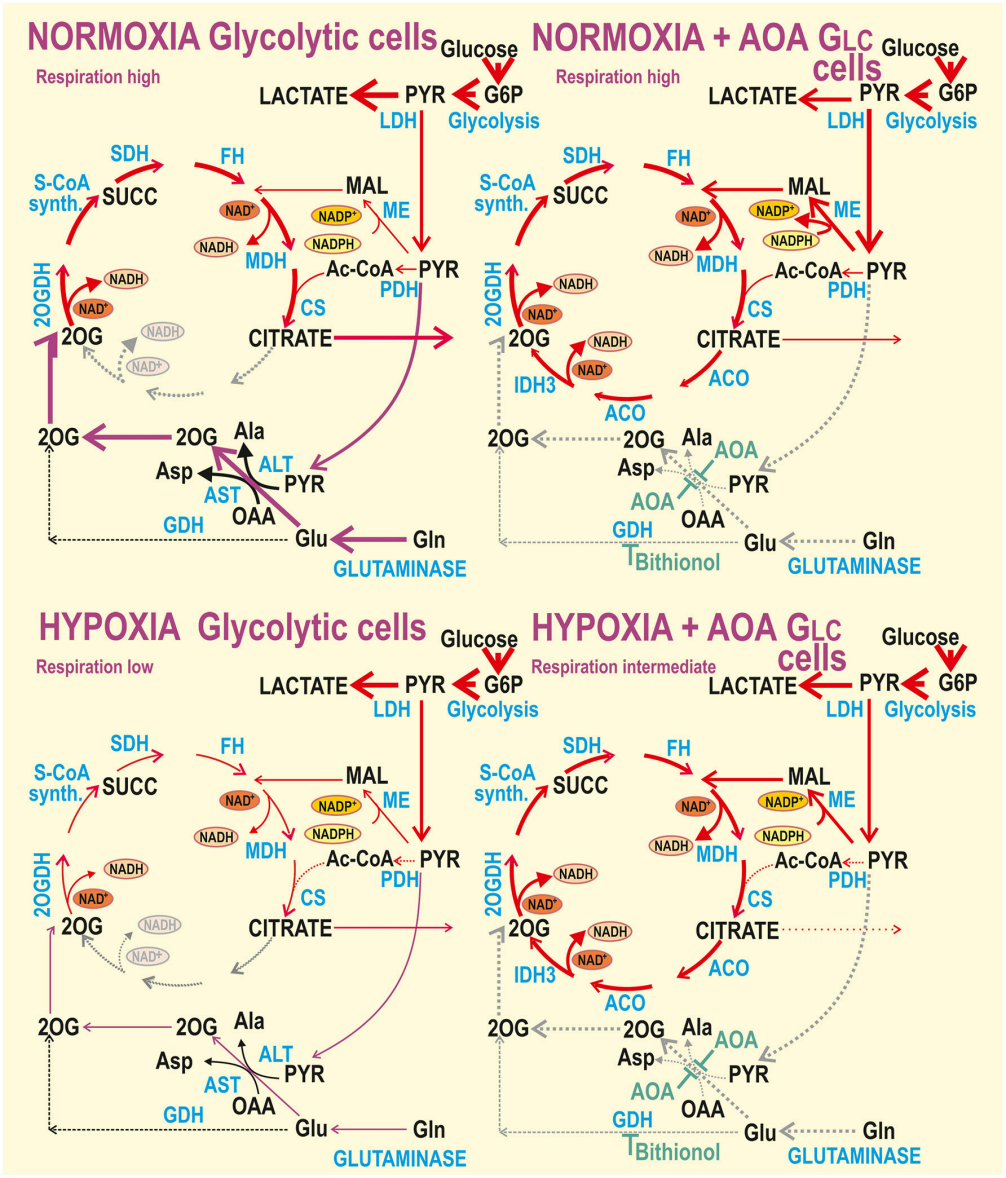
Schemes of normoxic *vs*. hypoxic metabolism for glycolytic cells with or without inhibited aminotransferases and GDH. Hypothetical interpretation of the obtained data for glycolytic cells is illustrated by thicker arrows representing higher metabolic fluxes or gray dotted arrows representing interrupted or inhibited fluxes. Abbreviations see Figure 7.

In contrast, the canonical HIF response exists in glycolytic cells (Figure 8). The HIF response in glycolytic cells leads to very high levels of PDH phosphorylation (37), causing insufficient acetyl-CoA entry into the Krebs cycle. Despite the resulting hypoxic inhibition of almost all PDH activity, hypoxia-adapted glycolytic HepG2 cells respire by rates accounting for ~40% of normoxic rates and leave the OXPHOS machinery partially dormant, but intact and ready to function (37). The intactness is manifested here by the addition of the cell-permeant 2-oxoglutarate derivative (dm2OG), which re-established respiration and mitochondrial ATP production.

Aminotransferases again play an important role for glycolytic cells, which are not so essentially dependent on glutaminolysis and OXPHOS at normoxia. This independence is reflected also by already much lower phosphorylating/non-phosphorylating respiration ratios in the presence of dm2OG at normoxia and by respiration independence on aminotransferase inhibition by AOA at hypoxia. As pointed out above, the HIF-mediated glycolysis upregulation is so intensive, that it does allow even higher respiration with AOA. This demonstrates a wide metabolic plasticity of the glycolytic cells (Figure 8). Also, overexpression of malic enzyme, frequent in tumor cells, was suggested to facilitate lower dependence on the glutaminolysis (58). Malate can be retained in mitochondria or exported to generate NADPH (necessary for biosynthetic pathways) by the mitochondrial or cytosolic malic enzyme, respectively. When retained, mitochondrial malic enzyme converts it to pyruvate. On the other hand, malate contributes to oxaloacetate production and oxaloacetate is used together with glutamine by AST for conversion to 2OG and aspartate.

Indeed, hypoxia-adapted glycolytic HepG2 cells exhibit saturated lactate formation, allowing the entry of a minimum amount of pyruvate into the Krebs cycle (37). The rate of OXPHOS at hypoxia is lower than half of that at atmospheric conditions (37). This is reflected also by less than half ATP levels (Figure 6). Evidently, glycolysis cannot compensate for the decreased capacity of OXPHOS. However, when the entry of 2OG to the different point of the Krebs cycle is ensured by the dm2OG presence during hypoxic adaptation, it does not restore OXPHOS as reflected by unchanged phosphorylating/non-phosphorylating respiration ratios, but is sufficient to restore the total ATP levels. This effect stems from the surplus substrate available.

Upon hypoxia cells recruit their resources to survive (55) and may employ cell to cell metabolic exchanges, thus responding to the fluctuating microenvironment. Cancer cells also control the overall redox state. The reduced glutathione fraction related to the total glutathione was drastically, at least 2-fold, depleted after our mild hypoxic adaptation. In hyperglycemic Glc25 cells, the fraction of reduced glutathione was even lower than in hypoxic aglycemic cells and was not further decreased at hypoxia. Probably the cells attained the highest possible oxidized state (the lowest reduced status). This was correlating with their highest normoxic NOX activity. Nevertheless, hypoxic Glc25 cells dropped this NOX activity as well as NAD(P)H/FAD ratios. The NOX activity was still higher in normoxic Glc5 cells when related to aglycemic Oxphos cells. After hypoxic adaptation it insignificantly increased in Glc5 cells but elevated >2-fold in Oxphos cells. Despite the faster hypoxic NADPH formation by G6P dehydrogenase, its even faster consumption by NOX leads to ~20% decrease in NAD(P)H/FAD ratios. In contrast to Glc25 cells, the moderate decrease is observed in hypoxic Glc5 cells, which are intermediately oxidized and exert also intermediate NOX activity.

In conclusion, we report on a non-canonical HIF pathway that is unable to down-regulate OXPHOS in hypoxic aglycemic HepG2 cells, in which G6P as a precursor for the HIF-enhanced glycolytic pathway is supplied by galactose metabolism (Leloir pathway). Aglycemic cells are thus switched after hypoxic adaptation to the galactolysis-supplied glycolysis followed by the complete Krebs cycle, including the aconitase-IDH3 segment, and OXPHOS. Since the Leloir pathway is slow and may give a lower ATP yield than the complete glycolysis, aglycemic cells posses a compromised high energy demand which can be satisfied only by glutaminolysis at normoxia. Glutaminolysis supports the pyruvate conversion by aminotransferases to 2OG, which subsequently enters into the Krebs cycle and maintains the cell normoxic respiration. Consequently, these cells can survive only when OXPHOS proceeds simultaneously.

In contrast, when glucose is available, such as for the glycolytic HepG2 cells, their Warburg phenotype is apparently further enhanced in hypoxia as a result of the typical, canonical, HIF pathway regulation. The Warburg phenotype represents the aerobic glycolysis with pyruvate channeling to lactate, and only a minor acetyl-CoA entry into the Krebs cycle. Nevertheless, at normoxia, respiration of glycolytic HepG2 cells can be surprisingly equal to respiration of aglycemic cells due to the pyruvate utilization by the alanine aminotransferase within the ongoing glutaminolysis. But neither this pathway nor glycolytic pyruvate substitute completely for the normoxic Krebs cycle turnover in hypoxia, as reflected by the declining respiration and OXPHOS intensity. In turn, the plasticity of glycolytic cells allows their respiration even at inhibited alanine aminotransferase and survival without glutamine.

In summary, we demonstrated a high metabolic flexibility of human hepatocarcinoma HepG2 cells challenged by a sole aglycemia or in combination with hypoxia. While glutaminolysis prevails at aglycemia, after adaptation to hypoxia glutamine no longer serves as the predominant energy substrate. Hypoxic reprogramming by the HIF system induces glycolysis, which can be fed by galactolysis (Leloir pathway) at aglycemia.

## Author contributions

All co-authors have designed experiments. LP-H and JJ conducted experiments, PJ analyzed data and wrote the article.

### Conflict of interest statement

The authors declare that the research was conducted in the absence of any commercial or financial relationships that could be construed as a potential conflict of interest.
